# Motorized Shoes Induce Robust Sensorimotor Adaptation in Walking

**DOI:** 10.3389/fnins.2020.00174

**Published:** 2020-03-06

**Authors:** Yashar Aucie, Xunjie Zhang, Randy Sargent, Gelsy Torres-Oviedo

**Affiliations:** ^1^Department of Bioengineering, Swanson School of Engineering, University of Pittsburgh, Pittsburgh, PA, United States; ^2^Nimbus Robotics, Pittsburgh, PA, United States; ^3^The Robotics Institute, School of Computer Science, Carnegie Mellon University, Pittsburgh, PA, United States

**Keywords:** locomotion, motor learning, rehabilitation robotics, real-world, portable device

## Abstract

The motor system has the flexibility to update motor plans according to systematic changes in the environment or the body. This capacity is studied in the laboratory through sensorimotor adaptation paradigms imposing sustained and predictable motor demands specific to the task at hand. However, these studies are tied to the laboratory setting. Thus, we asked if a portable device could be used to elicit locomotor adaptation outside the laboratory. To this end, we tested the extent to which a pair of motorized shoes could induce similar locomotor adaptation to split-belt walking, which is a well-established sensorimotor adaptation paradigm in locomotion. We specifically compared the adaptation effects (i.e. after-effects) between two groups of young, healthy participants walking with the legs moving at different speeds by either a split-belt treadmill or a pair of motorized shoes. The speeds at which the legs moved in the split-belt group was set by the belt speed under each foot, whereas in the motorized shoes group were set by the combined effect of the actuated shoes and the belts’ moving at the same speed. We found that the adaptation of joint motions and measures of spatial and temporal asymmetry, which are commonly used to quantify sensorimotor adaptation in locomotion, were indistinguishable between groups. We only found small differences in the joint angle kinematics during baseline walking between the groups – potentially due to the weight and height of the motorized shoes. Our results indicate that robust sensorimotor adaptation in walking can be induced with a paired of motorized shoes, opening the exciting possibility to study sensorimotor adaptation during more realistic situations outside the laboratory.

## Introduction

The motor system has the flexibility to update motor plans according to systematic changes in the environment or the body. This human ability is studied in the laboratory through sensorimotor adaptation paradigms imposing sustained and predictable motor demands specific to the task at hand, such as unusual visuomotor rotations (e.g. Krakauer et al., 2000) or constant forces during walking (Savin et al., 2010) or reaching (Shadmehr and Mussa-ivaldi, 1994). For example, split-belt walking is a well-established paradigm in which participants update spatiotemporal gait features in response to a persistent speed difference between their legs (Dietz et al., 1994; Reisman et al., 2005; Malone et al., 2012). Important motor adaptation principles have been learned from these sensorimotor adaptation paradigms, such as the computations underlying motor adaptation (Thoroughman and Shadmehr, 2000; Haruno et al., 2001; Smith et al., 2006) or neural structures involved in this process (Deuschl et al., 1996; Smith and Shadmehr, 2005; Morton and Bastian, 2006). However, there are inherent limitations to laboratory-based studies that bring into question the extent to which principles governing motor adaptation apply to motor learning in the real-world.

Specifically, there are task-constraints in laboratory-based studies that limit our ability to investigate factors that are critical for motor learning outside the laboratory setting. For example, laboratory-based protocols challenge the study of extended practice, which is a critical aspect of motor learning (Ericsson and Pool, 2016; Haith and Krakauer, 2018). There are several efforts to investigate the effect of extended practice on motor behavior by bringing participants to the laboratory multiple times (Day et al., 2018; Leech et al., 2018; Hardwick et al., 2019). This research effort would be facilitated if individuals could practice outside the laboratory setting. Further, we constrain movements by for example making people walk at a constant speed (Dietz et al., 1994), or repeatedly reach to a certain direction (Krakauer et al., 2000). This is done to simplify the control variables affecting the studied behavior, and at the extreme, this could yield to the study of unnatural behaviors, whose underlying mechanisms might not apply to realistic situations. A byproduct from task-constraints is the context-specificity of motor patterns learned in the laboratory that is movements adapted with the device only partially carry over to movements without the training device (Kluzik et al., 2008; Torres-Oviedo and Bastian, 2010). This is detrimental not only because it limits our capacity for studying the generalization of motor learning across distinct situations, but also because it limits the possibility for using laboratory-based tasks for motor rehabilitation. Notably, it is well-accepted that the generalization of motor patterns from trained to untrained situations can be improved when the two contexts are more similar to one another (Tulving and Thomson, 1973; Spear, 1978; Bouton et al., 1999). Thus, there could be more generalization of laboratory-based knowledge to realistic situations when the tasks studied in the laboratory are more similar to those observed under naturalistic conditions.

Portable devices may offer the possibility to overcome the limitations of laboratory-based studies of motor learning. For example, portable devices allow us to investigate motor learning in real-life settings, such as studies of surgical training with the same tools that are used at the clinic (Sharon et al., 2017). In addition, the portability of training devices also enables the study of extended practice since individuals are not constrained to only train in the laboratory setting (Hardwick et al., 2019). Further, portable devices might allow for more complex movements that involve the whole body (Haar et al., 2019), which might lead to greater motor variability – a key factor for motor learning (Kelly and Sober, 2014; Wu et al., 2014; Therrien et al., 2016). In the context of locomotion there have been efforts to develop portable devices to study motor adaptation (Handzic et al., 2011; Handzic and Reed, 2013; Lahiff et al., 2016). However, the previous devices were passive, lacking the control over the speed difference between the feet. In addition, gait adjustments induced by these devices are not as robust as the ones observed with laboratory-based apparatus such as split-belt treadmills. Thus, we asked if a pair of motorized shoes could induce locomotor adaptation comparable to split-belt walking, which is a well-established sensorimotor adaptation paradigm in locomotion.

We specifically hypothesized that introducing a speed difference between participant’s feet with the motorized shoes would result in adaptation of spatiotemporal gait patterns similar to split-belt walking. To test this hypothesis, we compared locomotor adaptation at comparable speed differences imposed by either a pair of motorized shoes or a split-belt treadmill. If the locomotor adaptation with the motorized shoes is similar to the one observed during split-belt walking paradigm, participants could start wearing these shoes outside the laboratory, which would offer the exciting possibility to study locomotor learning under more realistic situations.

## Materials and Methods

### Participants

We investigated if a pair of motorized shoes could induce locomotor adaptation and after-effects similar to a split-belt treadmill. To this end, a group of 18 young, healthy, and naïve adults were adapted using either (1) the motorized shoes that imposed speed differences between the feet using actuated wheels under the shoe (motorized shoes group: *n* = 9; three females: 26.6 ± 3.5 years) or (2) a split-belt treadmill, in which belts moved at different speeds (split-belt group: *n* = 9; four females: 25.3 ± 4.3 years). The Institutional Review Board at the University of Pittsburgh approved our experimental protocol and all participants gave their written informed consent before being tested.

### Set Up

**FIGURE 1:**
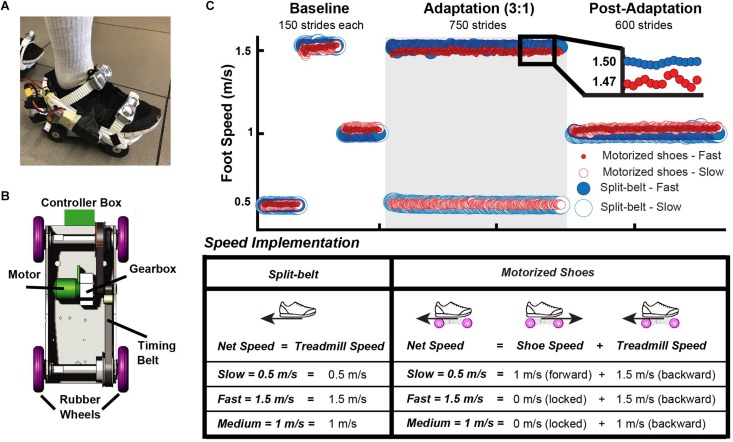
**(A)** A motorized shoe involving proprietary technology was used to induce adaptation in the motorized shoes group. **(B)** Schematic of the motorized shoe. This consists of a motor, a controller box, a gearbox, two toothed timing belts, and four rubber wheels. **(C)** Mean time courses for foot speed across participants for the motorized shoes and the split-belt groups. The white background indicates experimental epochs of “tied” walking when both feet moved at the same speed, whereas the gray background indicates the epoch of “split” walking when the dominant leg moved three times faster than the non-dominant leg. The table summarizes the procedure used to set the slow, fast, and medium speeds for each foot. The same procedure was used in all epochs. It is worth pointing out that the treadmill always moved at 1.5 m/s during adaptation in the motorized shoes group. The speed difference between feet was achieved by locking the wheels on the fast side and moving the slow foot forward at 1 m/s to obtain a net speed of 0.5 m/s on the slow side. Of note, the foot’s speed on the fast side was slightly slower on the motorized shoes than the split-belt group.

The motorized shoes group walked on the treadmill while wearing the custom made motorized shoes (Nimbus Robotics, Pittsburgh, PA, United States) as shown in Figure 1A on top of their normal walking shoes. In brief, the shoes were designed to move an individual (weighing <100 kg) up to 1 m/s in the forward direction only (i.e. wheels cannot be actuated to rotate backward). Each of the motorized shoe (∼1.7 kg) consisted of a motor, a controller box, a gearbox, two toothed timing belts, and four rubber wheels (Figure 1B). Lithium batteries (3V) were used to power the motor, which rotated the timing belts via a gearbox connecting the two. The feet moved at different speeds with the motorized shoes by locking the wheels of one foot and actuating the wheels of the other foot, such that the combined effect of the treadmill’s belt moving the foot backward and the motorized shoe moving the foot forward would result in the desired foot speed of 0.5 m/s (Figure 1B). To this end, the timing belts and rubber wheels were coupled to rotate the wheels such that they locked the non-actuated shoe during stance (∼0 m/s) and moved the actuated shoe forward at a linear speed of 1 m/s. The controller boxes received signals through a remote controller operated by the experimenter. All software for the controller boxes and the remote controller were written in Python. Details on the control software are published in Zhang (2017) and a detailed description of the motorized shoes will be revealed in the full utility patent (currently in provisional status). The split-belt group did not wear the motorized shoes and walked with their regular shoes on an instrumented split-belt treadmill (Bertec, Columbus, OH, United States).

### General Paradigm

All participants adapted following a conventional sensorimotor adaptation paradigm that consisted of three walking conditions: baseline, adaptation, and post-adaptation (Figure 1C, Top). During these periods, participants’ feet moved at one of three possible speeds: slow (0.5 m/s), medium (1 m/s), or fast (1.5 m/s). The implementation of these speeds is displayed in Figure 1C. Participants in the motorized shoes group wore these shoes throughout the experimental protocol, whereas participants in the split-belt group wore regular sneakers. Thus, the net foot speed in the motorized shoes group was the sum of the treadmill’s speed (moving the foot backward) and the shoe’s speed (moving the foot forward), whereas the foot speed in the split-belt group was only dependent on the treadmill’s speed (Figure 1C, Bottom). For example, in the motorized shoes group the slow foot speed (0.5 m/s) resulted from the combined effect of the treadmill moving the foot at 1.5 m/s (backward) and the motorized shoe moving the foot at 1 m/s (forward) (i.e. 1.5 −1 = 0.5 m/s). The motorized shoes were OFF and wheels were locked (0 m/s) at the fast and medium speeds; thus, the foot’s net seed at those velocities was only determined by the treadmill’s speed. This was done to maximize the experiment’s duration for a given battery life. Our approach also enabled us to implement the same feet speed’s in both groups while participants in the motorized shoes group walked on a regular treadmill (i.e. both belts moving at the same speeds).

A baseline period was collected during which both feet moved at either slow, fast, or medium speeds for 150 strides each (Figure 1C, Top). The baseline behavior during the slow and fast speeds served as a reference for the adaptation condition when the feet moved at different speeds, whereas the medium speed served as a reference for the post-adaptation period when the two feet move at the same medium speed. Moreover, the baseline speed was matched not only in the speed at which the feet moved, but also on how this speed was implemented. For example, in the motorized shoes group, the shoe was actuated in the slow side (net speed = 0.5 m/s) and it was OFF (wheels locked) in the fast side (net speed = 1.5 m/s) during the adaptation period. Accordingly, both motorized shoes were either actuated or OFF in the slow and fast baselines, respectively. The adaptation period lasted 750 strides (approx. 15 min) and the dominant leg (self-reported leg to kick a ball) walked fast. The speed difference and period duration was selected to match other split-belt walking studies showing robust gait adaptation (Sombric et al., 2019). Following the adaptation block, all participants experienced a post-adaptation period of 600 strides during which both feet moved at 1 m/s, which was the average speed of the fast and slow feet. The purpose of this phase was to measure the adaptation effects and its washout when the speed perturbation induced by different devices was removed.

### Data Collection

**FIGURE 2:**
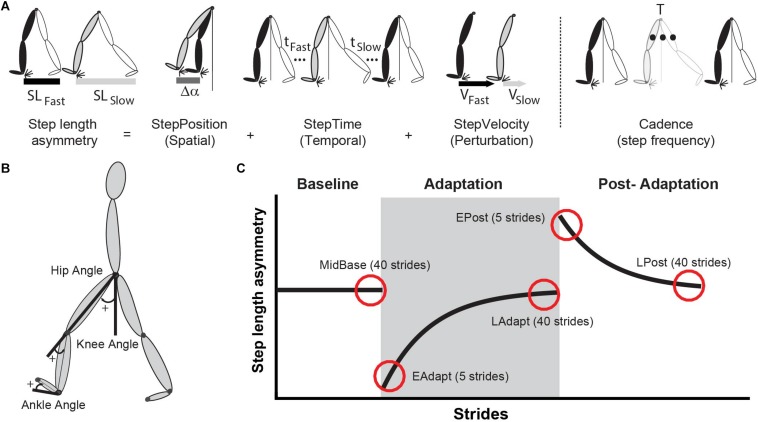
**(A)** This schematic illustrates step length asymmetry and its decomposition into StepPosition, StepTime, and StepVelocity. Step length asymmetry is quantified as the difference between fast and slow step lengths, normalized by stride length. The equation and decomposition are explained in detail in the section “Materials and Methods” of this manuscript. In brief, (StepPosition) differences between the fast (black leg) and the slow (gray leg) leading leg’s positions contribute to step length asymmetry. Similarly, differences in the trailing leg’s positions (white legs) also contribute to step length asymmetry. The trailing leg’s position depends on step time and step velocity. Consequently, differences in step times (*t*_fast_ and *t*_slow_) or step velocity (*V*_fast_ and *V*_slow_) leads to step length asymmetry. We also show a schematic of Cadence, which is computed as the inverse of the gait period (T). **(B)** Illustration of reflective marker positions and joint angle conventions. **(C)** Epochs of interest are illustrated by the red circles placed over a schematic of step length asymmetry. Shaded gray area represents the adaptation period when the feet move at different speeds (“split” walking), whereas white areas represent when the feet move at the same speed.

All participants walked on an instrumented treadmill either with or without the motorized shoes, while kinematic and kinetic data were collected to characterize participants’ gait. Kinematic data were collected at 100 Hz with a passive motion capture system (Vicon Motion Systems, Oxford, United Kingdom) and kinetic data were collected at 1000 Hz using force plates embedded in the treadmill. Gaps in raw kinematic data due to marker occlusion were filled by visual inspection of each participant in Vicon Nexus software. Positions from the toe (5^th^ metatarsal), ankle (lateral malleolus), knee (lateral epicondyles), and the hip (greater trochanter) were collected bilaterally (Figure 2B). Heel-strikes (i.e. foot landing) and toe-offs (i.e. foot lift off) were identified using the ground reaction force (Fz) perpendicular to the walking surface. More specifically, heel-strike was defined as the instance when Fz > 30 N and toe-off as the instance when Fz < 30 N. We used this force threshold to have equivalent event detection (i.e. heel strike and toe off) on the treadmill for both groups since each of the motorized shoe weighted 17 N (∼1.7 kg in mass).

### Data Analysis

We compared the gait pattern between the motorized shoes and split-belt groups in terms of spatial and temporal symmetry measures that are known to adapt on the split-belt treadmill (Figure 2A; Finley et al., 2015). Specifically, we used step length asymmetry as a robust measure of adaptation. Step length asymmetry was defined as the difference between step lengths (i.e. distance between ankles) with the slow leg vs. the fast leg (Eq. 1). A zero value of step length asymmetry indicated that both step lengths were equal and a positive value indicated that the step length of the fast (dominant) leg was longer than the slow (non-dominant) leg. Step length asymmetry was further decomposed into StepPosition, StepTime, and StepVelocity because these parameters have been shown to be adapted differently during split-belt walking (Finley et al., 2015). The StepPosition quantified the difference in positions of the leading leg (i.e. leg in front of the body) between two consecutive steps (Eq. 2). The StepTime quantified the difference in the duration of each of these steps (Eq. 3). Lastly, the StepVelocity quantified the difference in the velocities of each foot with respect to the body for these two steps (Eq. 4). Since participants take steps with different sizes, we normalized the differences in step length, StepPosition, StepTime, and StepVelocity by their stride length, quantified as the sum of two step lengths. This allowed us to avoid inter-subject variability. For visualization purposes, these parameters were smoothed with a five-step running average.

(1) $$S\,t\,e\,p\,l\,e\,g\,n\,t\,h\,a\,s\,y\,m\,m\,e\,t\,r\,y=\frac{F\,a\,s\,t\,S\,t\,e\,p\,L\,e\,n\,g\,t\,h-S\,l\,o\,w\,S\,t\,e\,p\,L\,e\,n\,g\,t\,h}{S\,L}$$

(2) $$S\,t\,e\,p\,P\,o\,s\,i\,t\,i\,o\,n=\frac{\left(\Delta \,\alpha_{f\,a\,s\,t}-\Delta \,\alpha_{s\,l\,o\,w}\right)}{S\,L}$$

(3) $$S\,t\,e\,p\,T\,i\,m\,e=\frac{\frac{v_{s\,l\,o\,w}+v_{f\,a\,s\,t}}{2}\,\left(t_{s\,l\,o\,w}-t_{f\,a\,s\,t}\right)}{S\,L}$$

(4) $$S\,t\,e\,p\,V\,e\,l\,o\,c\,i\,t\,y=\frac{\frac{t_{s\,l\,o\,w}+t_{f\,a\,s\,t}}{2}\,\left(v_{s\,l\,o\,w}-v_{f\,a\,s\,t}\right)}{S\,L}$$

In these equations, Δα indicates the difference between each foot’s position (i.e. ankle marker) and the body (i.e. mean position of the two hip markers) at ipsilateral heel strike (Figure 2A); In addition, *t* indicates the step time defined as the duration between the heel-strike of ipsilateral leg to the contralateral leg; and *v* indicates the step velocity quantified as the relative velocity of the foot with respect to the body. When walking on the treadmill, *v*_*slow*_ and *v*_*fast*_ approximated the speeds of the slow and fast belt, respectively. Therefore, StepVelocity was mostly reflective of belt speed difference, rather than participants’ behavior. Finally, note that all measures were normalized by each participant’s stride length (SL, sum of both step lengths) to account for inter-subject differences in step sizes.

We also computed joint angles and cadence to determine the impact of the motorized shoes on each foot’s motion and step frequency. Ankle, knee, and hip angles were computed on the sagittal plane (2D) to directly contrast our results to previous reports of joint angles during split-walking (Reisman et al., 2005). Joint angles were calculated such that flexion/dorsiflexion was positive and extension/plantarflexion was negative (Figure 2B). We also defined all angles to have value of 0° at the neutral standing position (i.e. full extension for knee and hip and approximately 90° angle between shank and foot for the ankle). More specifically, ankle angles were calculated as the angle between the foot (ankle marker to toe marker vector) and the shank (ankle marker to knee marker vector) subtracted from each participant’s neutral position (i.e. mean and standard deviation: 88.4 ± 3.7° for the group wearing the motorized shoes and 91.2 ± 0.95° for the split-belt group). Knee angles were calculated as the angle between the shank and the thigh (knee marker to hip marker vector) subtracted from 180°. Lastly, we computed the hip angles as the angle between the thigh and the vertical unit vector. Angle data was time-aligned and binned to compute mean angle values over six intervals of interest during the gait cycle. This was done to focus on changes in angles within the gait cycle, rather than on changes due to differences in cycle duration across the distinct walking conditions (Dietz et al., 1994; Reisman et al., 2005). More specifically, we computed averaged angle values over six phases of interest (Perry, 2010): double support (DS1 and DS2), single stance (SS1 and SS2), and the swing phases (SW1 and SW2). Double support during early stance (DS1) was defined as the period from heel strike to contralateral toe off. Single stance (from contralateral toe-off to contralateral heel strike) was divided into two equal phases (SS1 and SS2). Double support during late stance (DS2) was defined as the interval from contralateral heel strike to ipsilateral toe off. Finally, the swing phase (from ipsilateral toe-off to ipsilateral heel-strike) was divided into two equal phases (SW1 and SW2). Joint angles were assessed in eight participants per group since the remaining two participants (one per group) was missing essential marker data. Lastly, we computed cadence (i.e. number of strides per second) to determine if this gait feature was altered by wearing the motorized shoes.

### Outcome Measures

Each gait parameter was analyzed during four experimental epochs of interest (early adaptation, late adaptation, early post-adaptation, and late post-adaptation) to compare the adaptation and after-effects between the motorized shoes and the split-belt treadmill groups. We computed the averaged value of each parameter over these epochs as follows. First, we removed the five strides at the beginning and at the end of each trial to eliminate effects of holding on to the handrail when starting and stopping the treadmill. This was done to characterize people’s movement when no individuals were holding on to the safety rail. Then, we computed the average value for each epoch as follows: early adaptation (EAdapt, average of five strides: 6^th^–10^th^ stride), late adaptation (LAdapt, average of 40 strides: 706^th^–745^th^ stride), early post-adaptation (EPost, average of five strides: 6^th^–10^th^ stride), and late post-adaptation (LPost, average of 40 strides: 546^th^–595^th^ stride) (Figure 2C). All of the parameters were corrected by any baseline biases (MidBase, average of 40 strides: 106^th^–145^th^ stride). EAdapt gave us information about the induced perturbation by the “split” condition, while the LAdapt provided information regarding the steady-state behavior at the end of the adaptation trial. The behavior during EPost was quantified to assess how much participants adapted to the new walking pattern (e.g. after-effects). Finally, we assessed LPost behavior to ensure that participants returned to their baseline walking behavior (e.g. washout). Moreover, we used joint angle measures to determine the effect of the motorized shoes on the overall gait pattern. This analysis was intended to determine if participants were actually walking with the motorized shoes (i.e. not dragging their feet or sliding their feet). To this end, we computed the averaged value over the last 40 strides (after removing the very last five strides, as in the other kinematic parameters) for each one of the four experimental epochs of interest (i.e. SBase, FBase, MidBase, and LAdapt).

### Statistical Analysis

We performed one-sample Kolmogorov–Smirnov tests to determine if each parameter (i.e. Step length asymmetry, Step lengths, StepPosition, StepTime, StepVelocity, and Cadence) was normally distributed in every epoch of interest (i.e. EAdapt, LAdapt, EPost, and LPost). We found that all parameters were normally distributed, thus we ran separate two-way repeated measures ANOVAs to test the effects of epochs and groups (i.e. motorized shoes vs. split-belt) on each of our gait parameters. Statistical analysis was done with unbiased data (i.e. MidBase was subtracted from all the epochs) to focus on changes that occurred beyond those due to distinct group biases. In case of significant main or interaction effects, we used Fisher’s *post hoc* testing to determine whether values were different between groups. We chose this *post hoc* testing to be more sensitive to potential group differences. Lastly, we performed a one-sided one sample *t*-test to determine whether early post-adaptation values were different from zero. This was done to determine if after-effects were significant in each group. Comparisons between post-adaptations values across groups were only done when we found significant interactions between group and epoch.

Two separate multiple linear regressions were performed to determine if the individual variation in two independent variables: (1) StepPosition and (2) StepTime in late adaptation could be predicted by two regression coefficients and their interaction: group (categorical factor), StepVelocity (continuous variable), and group#StepVelocity (interaction). We also performed two separate multiple linear regressions to determine if the individual variation in after-effects in StepPosition and StepTime (two independent variables) were predicted by group or each respective steady state (StepPosition LAdapt or StepTime LAdapt). This was done because we observed speed differences between the groups (Figure 1C, Top) that could impact the extent of adaptation and after-effects on spatial and temporal measures.

Joint angles were compared across groups using unpaired *t*-test for each of the gait phases. We reasoned this was an appropriate statistical test to compare the behavior across groups given that joint angles are highly temporally correlated within the gait cycle and spatially correlated across segments. We subsequently corrected the significance threshold for each epoch using a Benjamini–Hochberg procedure (Benjamini and Hochberg, 1995), setting a false discovery rate of 5% (FDR correction). The reason for choosing this correction was due to higher number of comparisons that we made.

A significance level of α = 0.05 was used for all statistical tests. Stata (StataCorp., Collage Station, TX, United States) was used to perform the ANOVAs, whereas MATLAB (The MathWorks, Inc., Natick, MA, United States) was used for all other analyses.

## Results

### Motorized Shoes Can Induce Robust Sensorimotor Adaptation of Locomotion

**FIGURE 3:**
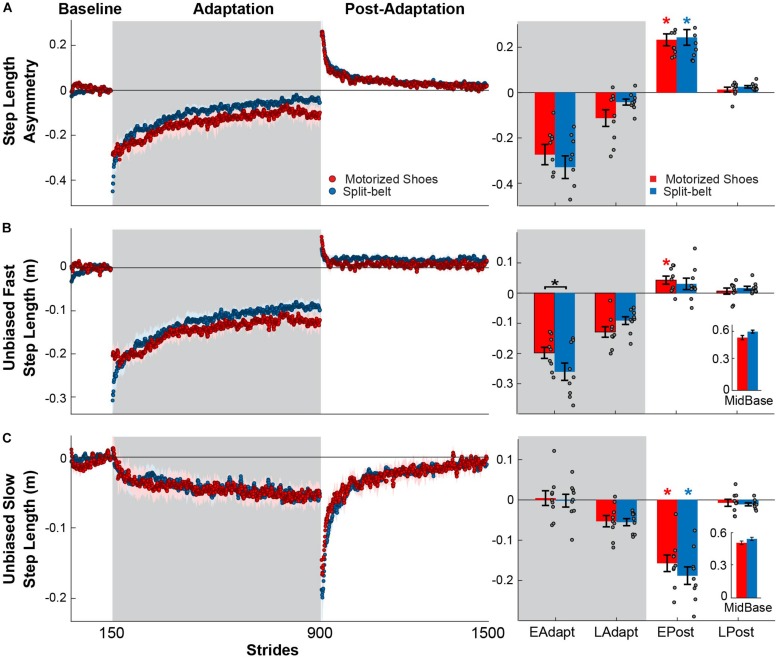
Modulation of step length asymmetry and step lengths. (**A–C**, Left panel) Time courses for step length asymmetry and individual step lengths during medium baseline, adaptation, and post-adaptation. Shaded gray area represents the adaptation period when the feet move at different speeds (“split” walking), whereas white areas represent when the feet move at the same speed. Colored dots represent the group average of five consecutive strides and colored shaded regions indicate the standard error for each group (motorized shoes: red; split-belt: blue). (**A–C**, Right panel) Bar plots indicate the mean ± standard errors for step length asymmetry and step lengths for each group and epoch of interest. Note that the reported step lengths are unbiased. This was done by subtracting the averaged step length values during baseline at medium speed in each participant. Significant differences for *post hoc* tests were indicated as follows. Black asterisks over the bracket above each epoch represent statistical significant differences between the motorized shoes and the split-belt groups (*p* < 0.05). Colored asterisks over the bars indicate significant after-effects (i.e. early post-adaptation is significantly different from baseline; *p* < 0.05) for each of the groups (motorized shoes: red; split-belt: blue). The small bar plots on the right indicate the mean ± standard errors for the step lengths for each group during medium baseline.

Our results show that the motorized shoes were able to induce similar adaptation of step length asymmetry compared to the split-belt treadmill. Specifically, there were no significant group (*F*_(__1_,_48__)_ = 0.21, *p* = 0.65) or group by epoch interaction effects (*F*_(__3_,_48__)_ = 1.26, *p* = 0.29) on the adaptation of step length asymmetry, indicating that this parameter was similarly modulated throughout the experiment between the motorized shoes and split-belt groups (Figure 3A). We observed a significant main effect of epoch (*F*_(__3_,_48__)_ = 94.91, *p* < 0.001) in step length asymmetry and found that both groups had significant after-effects (motorized shoes: *p* < 0.001; split-belt: *p* < 0.001; Figure 3A). While modulation of step length asymmetry was indistinguishable between groups, we observed small differences in the adaptation of the fast leg’s step length. Specifically, we found a group by epoch interaction effect in the fast step length (*F*_(__3_,_48__)_ = 3.18, *p* = 0.032; Figure 3B) driven by between-group differences during the early adaptation phase (*p* = 0.012). While significant, this between-group difference might not be meaningful given that the values that observed in both groups fall within the range of those previously reported (Sombric et al., 2019). Moreover, after-effects in this parameter were significant in the motorized shoes group (*p* = 0.013), but not in the split-belt group (*p* = 0.15). In contrast, the adaptation of the slow leg’s step length was similar across groups throughout the experiment (group: *F*_(__1_,_48__)_ = 0.63, *p* = 0.44; group by epoch interaction: *F*_(__3_,_48__)_ = 0.69, *p* = 0.49; Figure 3C). We only found a significant epoch effect on slow step length (*F*_(__3_,_48__)_ = 70.47, *p* < 0.001) and substantial after-effects in both groups (motorized shoes: *p* < 0.001; split-belt: *p* < 0.001). In summary, fast leg’s step length exhibited small differences between the motorized shoes and split-belt groups that did not impact the adaptation of step length asymmetry, which was indistinguishable between these groups.

### Smaller Speed Difference With the Motorized Shoes Reduced the Adaptation of StepPosition

**FIGURE 4:**
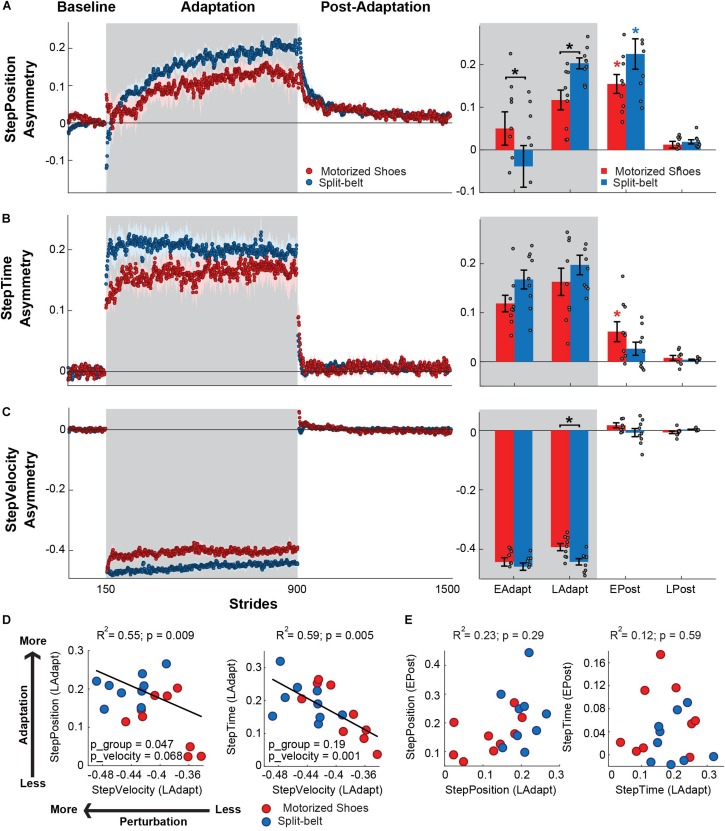
Adaptation of spatiotemporal components of step length asymmetry. (**A–C**, Left panel) Time courses for StepPosition, StepTime, and StepVelocity before, during, and after adaptation. Shaded gray area represents the adaptation period when the feet move at different speeds (“split” walking), whereas white areas represent when the feet move at the same speed. Colored dots represent the group average of five consecutive strides and colored shaded regions indicate the standard error for each group (motorized shoes: red; split-belt: blue). (**A–C**, Right panel) The bar plots indicate the mean ± standard errors for StepPosition, StepTime, and StepVelocity for each group and epoch of interest. Gray dots represent individual participants. Note that the values were corrected for baseline biases. Significant differences for *post hoc* tests were indicated as follows. Black asterisks over the bracket above each epoch represent statistical significant differences between the motorized shoes and the split-belt groups (*p* < 0.05). Colored asterisks over the bars indicate significant after-effects (i.e. early post-adaptation is significantly different from baseline; *p* < 0.05) for each of the groups (motorized shoes: red; split-belt: blue). **(D)** Scatter plots illustrate the association between the StepVelocity at steady state and either the StepPosition or StepTime at steady-state during adaptation (i.e. LAdapt). We present the *p*-values for the multiple regression model (p), for the continuous variable (StepVelocity, p_velocity) and for the categorical variable (group, p_group). **(E)** Scatter plots illustrate the association between the LAdapt and EPost for StepPosition and StepTime. No significant relations were observed for neither StepPosition nor StepTime.

We observed between-group differences in the adaptation of StepPosition (quantifying spatial asymmetry), but not StepTime (quantifying temporal asymmetry). This was indicated by the significant group by epoch interaction found in StepPosition (*F*_(__3_,_48__)_ = 3.47, *p* = 0.023), but not in StepTime (*F*_(__3_,_48__)_ = 2.39, *p* = 0.09) (Figure 4). *Post hoc* analyses indicated that these differences in StepPosition were driven by distinct early and late adaptation values of this parameter in the motorized shoes group compared to the split-belt group (early adaptation: *p* = 0.031; late adaptation: *p* = 0.036). Yet, after-effects in StepPosition were significant in both groups (motorized shoes: *p* < 0.001; split-belt: *p* < 0.001) and after-effects in StepTime were only significant in the motorized shoes group (motorized shoes: *p* = 0.017; split-belt: *p* = 0.087) Interestingly, we also found a group effect (*F*_(__1_,_48__)_ = 6.58, *p* = 0.021) on StepVelocity and a group by epoch interaction trending effect (*F*_(__1_,_48__)_ = 2.78, *p* = 0.051) (Figure 4C). In particular, the StepVelocity was smaller in the group with motorized shoes than in the split-belt group during late adaptation (*p* = 0.001), which we thought could impact the motor adaptation of the motorized shoes group. Thus, we performed multiple linear regression analysis on the late adaptation epoch with either StepTime or StepPosition as the dependent variable and StepVelocity as the predictor. StepVelocity was indeed related to StepTime (*R*^2^ = 0.59; *p* = 0.005; StepTime = −1.19 ^∗^ StepVelocity − 0.32) and StepPosition (*R*^2^ = 0.55; *p* = 0.009; StepPosition = −0.82 ^∗^ StepVelocity − 0.15). However, individual StepVelocity values were only a predictor of StepTime values [Group: p_group = 0.19, regression coefficient = 0.44, 95% CI = (−0.25, 1.13); StepVelocity: p_velocity = 0.001, regression coefficient = −1.99, 95% CI = (−3.08, −0.91); Interaction: p_group#velocity = 0.16, regression coefficient = 1.14, 95% CI = (−0.49, 2.78)], whereas the relation between StepVelocity and StepPosition was driven by a group effect (Group: p_group = 0.047, regression coefficient = 0.71, 95% CI = (0.0092, 1.4); StepVelocity: p_velocity = 0.068, regression coefficient = −1.01, 95% CI = (−2.1, 0.086); Interaction: p_group#velocity = 0.069, regression coefficient = 1.5, 95% CI = (−0.13, 3.16)] (Figure 4D). We also found that the inter-subject variability in steady-state values was not associated to individual after-effects in neither StepPosition (*R*^2^ = 0.23; *p* = 0.29), nor StepTime (*R*^2^ = 0.12; *p* = 0.59) (Figure 4E). To sum up, the reduced speed difference in the motorized shoes group limited the adaptation of StepPosition, but we still observed group after-effects with the motorized shoes in the spatial and temporal domains.

### Similar Cadence Is Observed Between the Groups Throughout the Experiment

**FIGURE 5:**
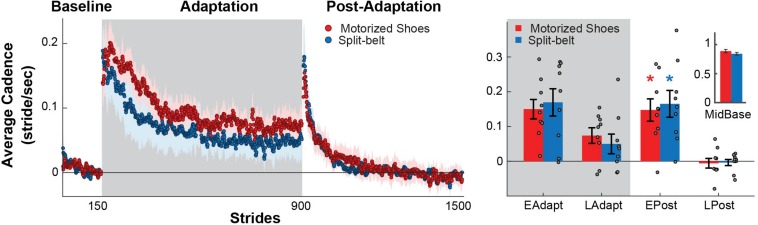
Modulation of cadence. **(Left)** Time courses during medium baseline, adaptation, and post-adaptation for the average cadence is shown for each group. Shaded gray area represents the adaptation period when the feet move at different speeds (“split” walking), whereas white areas represent when the feet move at the same speed. Colored dots represent the group average of five consecutive strides and colored shaded regions indicate the standard error for each group (motorized shoes: red; split-belt: blue). **(Right)** Bar plots indicate the mean ± standard errors for cadence for each group and epoch of interest. Note that the values were corrected for baseline biases (i.e. MidBase). Colored asterisks over the bars indicate significant after-effects (i.e. early post-adaptation is significantly different from baseline; *p* < 0.05) for each of the groups (motorized shoes: red; split-belt: blue). The small bar plot on the right indicates the mean ± standard errors for the Cadence for each group during medium baseline.

We found that the motorized shoes did not alter the modulation of cadence throughout the experiment compared to split-belt walking (Figure 5, left). Specifically, there were no significant group (*F*_(__1_,_48__)_ = 0.02, *p* = 0.88) or group by epoch interaction effects on cadence (*F*_(__3_,_48__)_ = 0.32, *p* = 0.81), indicating that the adaptation and after-effects of cadence were similar between groups (Figure 5, right). We also found that both groups exhibited increased cadences during early post-adaptation compared to baseline (motorized shoes: *p* = 0.002; split-belt: *p* = 0.003). In sum, individual wearing the motorized shoes modulate cadence similarly to individuals in the split-belt group.

### Effect of Wearing Motorized Shoes on Gait Kinematics

**FIGURE 6:**
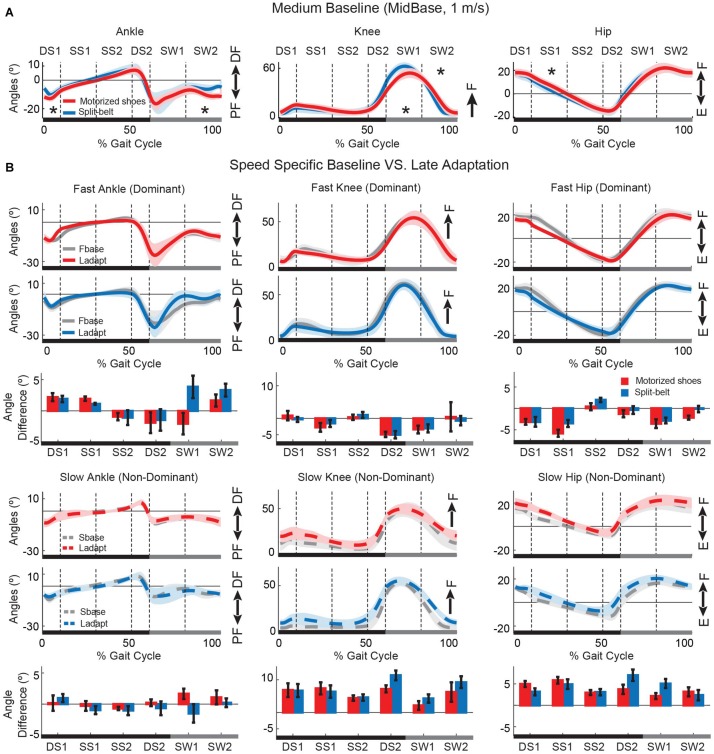
Joint angles over the gait cycle during baseline and adaptation. **(A)** Baseline joint angles are shown for the group walking with regular sneakers (i.e. blue trace) and the group walking with the motorized shoes (i.e. red trace). Solid lines represent the group average and shaded areas represent standard errors. Asterisks indicate instances during the gait cycle when joint angles were significantly different across groups. The overall motion for all joints was similar across groups, but hip flexion, knee flexion, and ankle dorsiflexion were smaller when wearing the motorized shoes. **(B)** Speed specific baseline (gray) and steady-state angle trajectories during adaptation for the motorized shoes (red) and the split-belt (blue) groups. Solid lines represent the motion of the leg walking fast in the split condition (colored lines) and in the fast baseline (gray) condition. The dashed lines represent the motion of the leg walking slow in the split condition (colored lines) and in the slow baseline (gray) condition. The bars represent the change from the speed-specific baseline to late adaptation in joint angles during different phases of the gait cycle. DS, double support; SS, single stance; SW, swing; DF, dorsiflexion; PF, plantarflexion; F, flexion; E, extension.

Overall, the gait pattern with and without the motorized shoes was similar. Figure 6A illustrates the joint angles over the gait cycle for the ankle, knee, and hip joints for the group wearing the motorized shoes (red) and the group wearing regular shoes (blue) during medium baseline walking. We found joint angles were the same between groups for most phases of the gait cycle, in which significance was determined with an FDR controlling procedure (18 comparisons, *p* > Pthreshold, Pthreshold = 0.0055, see the section “Materials and Methods”) (Figure 6A). There were only a few differences in specific phases of the gait cycle. Specifically, the motorized shoes group demonstrated reduced ankle dorsiflexion following ipsilateral heel strike and during late swing (double support DS1: *p* = 0.004, effect size = 3.3°; late swing SW2: *p* = 0.004, effect size = 4.1°). Moreover, the motorized shoes group exhibited reduced knee flexion compared to the split-belt group during early swing (SW1: *p* = 0.004, effect size = 7.8°), followed by slightly more knee extension in late swing (SW2: *p* = 0.001, effect size = 9.6°). Lastly, the motorized shoes group had larger hip flexion during stance of baseline walking (*p* = 0.005, effect size = 4.1°). While these between-group differences were significant, they should be interpreted consciously given the reliability of kinematic measurements. Namely, one can find significant changes in joint angles that are greater than 5° when measured across sessions within the same cohort of healthy, young participants (Wilken et al., 2012). Therefore, the differences that we find, ranging from 3.3° to 9.6°, might not be meaningful. In addition to baseline joint kinematics, we also compared late adaptation kinematics across groups (Figure 6B). Specifically, we contrasted the changes in joint angles during late adaptation relative to the speed-specific baseline for each of the six phases of the gait cycle. We found no differences between the groups (36 comparisons, *p* > Pthreshold), suggesting that joint angles were modulated similarly in the split condition with the motorized shoes or the split-belt treadmill. Thus, our results demonstrated that walking with the motorized shoes had only minor effects on joint kinematics and did not alter the adaptation of individual joint angles during split walking.

## Discussion

### Summary

We investigated if a pair of motorized shoes could induce split-like locomotor adaptation. We found that the adaptation effects induced by the motorized shoes moving at different speeds were as robust as those observed with a split-belt treadmill. Moreover, we found that the gait pattern was largely similar between walking with the motorized shoes or on the split-belt treadmill. Specifically, step length asymmetry, cadence, and step lengths were similar across groups during and after the split condition with either device. We only observed subtle differences in individual joint angles during the baseline condition with the motorized shoes compared to walking with regular shoes, which might be due to the greater height and weight of the motorized shoes. Taken together, our results suggest motorized shoes can induce robust sensorimotor adaptation in locomotion, opening the exciting possibility to study locomotor learning under more realistic situations outside the laboratory setting.

### Similar Walking and Adaptation With Split-Belt Treadmill and With Motorized Shoes

We demonstrated that the motorized shoes can induce locomotor adaptation largely similar to the adaptation induced with the split-belt treadmill. This was shown by the comparable adaptation across groups of gait parameters, such as step length asymmetry, and the same modulation of joint angles from baseline to adaptation for both groups. Namely, the initial and steady state values during the split condition for the split-belt group and motorized shoes group were consistent with values previously reported for joint angle kinematics (Winter, 1987; Reisman et al., 2005) and asymmetries in step length (Malone and Bastian, 2010; Finley et al., 2015), step position (Sombric et al., 2017), and step time (Gonzalez-Rubio et al., 2019). We found between-group differences in the fast step length during early adaptation, such that participants with the motorized shoes placed the fast leg closer to the body. This distinct behavior might also be explained by the fact that the balance is perturbed in the beginning of the split condition (Buurke et al., 2018; Iturralde and Torres-Oviedo, 2019) and it might be further challenged when stepping with the motorized shoes by augmenting the center of mass’ height, increasing even further gait instabilities while walking. However, this between-group differences might not be very meaningful and should be interpreted cautiously given than the range of these step length values fall within those previously reported (Sombric et al., 2019).

Participants with the motorized shoes reached lower steady state values of StepPosition (spatial) and slightly lower steady state values of StepTime (temporal) relative to the split-belt group. Our multiple regression analysis indicated that smaller speed differences (i.e. perturbation) were predictive of smaller steady state values for StepTime, but not StepPosition. Thus, perturbation size regulated the extent to which participants adapted in our temporal measure, as observed in other sensorimotor adaptation protocols of reaching (Morehead et al., 2015; Marinovic et al., 2017) or walking (Finley et al., 2015; Yokoyama et al., 2018). We did not find a direct relation between perturbation size and the reached steady state of StepPosition at an individual level, indicating that there are other factors, such as navigation strategies (Matthis et al., 2017) or practice (Day et al., 2018), influencing “where” people place their feet. Despite the subtle differences during adaptation, we saw similar after-effects between groups during early post-adaptation in all gait parameters. For example, cadence exhibited comparable changes between the groups during early adaptation and early de-adaptation, which is consistent with previous literature showing that stride time (i.e. inversely related to cadence) decreases in the beginning of adaptation (Reisman et al., 2005) and post-adaptation (MacLellan et al., 2014). In summary, our portable device induced significant adaptation and after-effects of gait asymmetries in space and time opening the door for studying locomotor adaptation outside of the laboratory.

We did not find a direct correspondence between adaptation and after-effects in neither the spatial nor the temporal domains. The positive relation between steady state values and after-effects is commonly found in reaching or saccadic movements with well-defined performance errors (Chen-Harris et al., 2008). This relation between steady-state values during the adaptation period and after-effects is, however, elusive in split-belt protocols. For example, gait parameters such as StepTime asymmetry can change dramatically during the Adaptation period (i.e. split condition) without showing any significant after-effects (Long et al., 2015; Gonzalez-Rubio et al., 2019). A recent study has also shown that changes in motor patterns during steady state split-belt walking and post-adaptation are not related and might be mediated by different neural substrates (de Kam et al., 2020). Taken together our findings further support the idea that gait adjustments during and after split-belt walking are governed by different mechanisms.

### Study Implications

We found a few differences in joint motions when walking with our motorized shoes during regular walking, which will be useful for future designs of this portable device. Notably, we observed gait changes during baseline walking (i.e. both feet moving at the same speed) with the motorized shoes that were consistent with other studies showing that shoe weight (Ochsmann et al., 2016) and height (McDonald et al., 2019) alter walking movements. In addition, the rigidity of the motorized shoes’ soles (Chiou et al., 2012) is another factor that might contribute to the differences that we observed in joint angles during baseline walking. Thus, our gait analysis enabled us to identify key shoe features that we will modify to reduce the effect of the motorized shoes on the regular walking pattern. This is important because contextual differences when wearing the motorized shoes could limit the extent of generalization of movements from walking with them to walking without this portable device. Locomotor adaptation with the motorized shoes overground could certainly reduce context-specific difference that limit the generalization of treadmill movements, such as visual flow (Torres-Oviedo and Bastian, 2012), walking speed (Dingwell et al., 2001), and step initiation. However, it remains to be determined whether contextual cues due to the height, weight, and rigidity of the motorized shoes would also limit the generalization of locomotor learning with them.

It is worth emphasizing that both groups were tested on a treadmill. This was done to track the movements of participants throughout the experiment, which we could not do with the motorized shoes outside the laboratory. Nevertheless, our results are promising because body-worn sensors, also referred to as wearables, now provide an inexpensive opportunity for the continuous monitoring of ambulatory activity in free-living environments (Wang and Adamczyk, 2019), which is a match to our technology. The actuation of the motorized shoes can add up to 1 m/s to the speed of each foot. Thus, we are certain that we can evoke speed differences comparable to split-belt studies (Reisman et al., 2005; Sombric et al., 2019) with these motorized shoes while walking over ground. In sum, the combination of these technologies can enable gait adaptation studies in realistic settings outside the laboratory. However, future studies with systems including adequate sensing mechanisms are needed to test this possibility.

Our results are also exciting because this portable device could also offer the possibility to study gait under more realistic situations, such as walking with self-regulated and variable gait speeds. It is well-accepted that motor variability can impact motor learning (Wu et al., 2014; Ulman et al., 2019), and walking on a treadmill is less variable compared to overground walking (Dingwell et al., 2001). Thus, having a device that can induce locomotor adaptation overground would help us gain more understanding about the relationship between variability and motor adaptation in walking. Moreover, learning a new task involves generation of new neural activity patterns, which appears after several days of practice (Oby et al., 2019). Our device will enable training over longer periods of time because individuals will be able to train at home and gain much more practice in the altered split environment than what is currently available. This can help us contribute to recent efforts to investigate the effect of long-term practice (Hardwick et al., 2019).

There have been efforts to develop portable rehabilitation devices (Handzic et al., 2011; Afzal et al., 2015; Lahiff et al., 2016; Calabrò et al., 2018) and assistive devices (Rao et al., 2008; Awad et al., 2017; Bae et al., 2018) to improve walking patterns in individuals with gait asymmetries, such as individuals post-stroke. While these apparatus could reduce the metabolic cost associated to gait in this clinical population (Awad et al., 2017) and improve walking speed (Rao et al., 2008; Buesing et al., 2015; Calabrò et al., 2018), these devices were unsuccessful in modifying the step length asymmetry (Handzic et al., 2011), which is an important parameter in rehabilitation of post-stroke patients (Patterson et al., 2008, 2014). For example, Lahiff et al. (2016) were able to modify push-off and breaking forces, but their device was unable to change step length of the participants. Similarly, Handzic and colleagues designed a device to passively induce a speed difference between the feet (Handzic et al., 2011; Handzic and Reed, 2013). However, this passive device induced limited changes in step length asymmetry post-adaptation (i.e. ∼5% of the after-effect size observed with the split-belt treadmill and motorized shoes). In sum, our study indicates that motorized shoes could tackle previous limitations altering gait asymmetries with portable devices and thus could be potentially used to correct asymmetric steps post-stroke.

## Data Availability Statement

The datasets generated for this study are available on request to the corresponding author.

## Ethics Statement

The studies involving human participants were reviewed and approved by the University of Pittsburgh Institutional Review Board. The patients/participants provided their written informed consent to participate in this study.

## Author Contributions

YA was involved in the acquisition, analysis, and interpretation of the data, drafting the work, and agreement to be accountable for all aspects of the work. XZ and RS were involved in the development of the motorized shoes and providing technical expertise for using the motorized shoes. GT-O was involved in the conception and design of the work, revising the work, and agreement to be accountable for all aspects of the work. All authors contributed to revising the manuscript and providing a final approval of the version to be published.

## Conflict of Interest

XZ was employed by the company Nimbus Robotics. The remaining authors declare that the research was conducted in the absence of any commercial or financial relationships that could be construed as a potential conflict of interest.
